# Regucalcin confers resistance to amyloid‐β toxicity in neuronally differentiated PC12 cells

**DOI:** 10.1002/2211-5463.12374

**Published:** 2018-01-20

**Authors:** Tomiyasu Murata, Masayoshi Yamaguchi, Susumu Kohno, Chiaki Takahashi, Mitsumi Kakimoto, Yukiko Sugimura, Mako Kamihara, Kiyomi Hikita, Norio Kaneda

**Affiliations:** ^1^ Laboratory of Analytical Neurobiology Faculty of Pharmacy Meijo University Nagoya Japan; ^2^ Department of Pathology and Laboratory Medicine David Geffen School of Medicine University of California, Los Angeles (UCLA) CA USA; ^3^ Division of Oncology and Molecular Biology Cancer Research Institute Kanazawa University Ishikawa Japan

**Keywords:** Alzheimer's disease, amyloid‐β, apoptosis, mitochondrial dysfunction, reactive nitrogen species, reactive oxygen species, regucalcin

## Abstract

Amyloid‐β (Aβ), a primary component of amyloid plaques, has been widely associated with the pathogenesis of Alzheimer's disease. The Ca^2+^‐binding protein regucalcin (RGN) plays multiple roles in maintaining cell functions by regulating intracellular calcium homeostasis, various signaling pathways, and gene expression systems. Here, we investigated the functional role of RGN against Aβ‐induced cytotoxicity in neuronally differentiated PC12 cells. Overexpression of RGN reduced Aβ‐induced apoptosis by reducing mitochondrial dysfunction and caspase activation. It also attenuated Aβ‐induced reactive oxygen species production and oxidative damage and decreased Aβ‐induced nitric oxide (NO) overproduction, upregulation of inducible NO synthase by nuclear factor‐κB, and nitrosative damage. Interestingly, the genetic disruption of RGN increased the susceptibility of neuronally differentiated PC12 cells to Aβ toxicity. Thus, RGN possesses antioxidant activity against Aβ‐induced oxidative and nitrosative stress and may play protective roles against Aβ‐induced neurotoxicity in Alzheimer's disease.

**Abbreviations**
$O_{2}^{-}$: superoxide
Aβ: amyloid‐β
iNOS: inducible nitric oxide synthase
NF‐κB: nuclear factor‐κB
NGF: nerve growth factor
NO: nitric oxide
ONOO^−^: peroxynitrite
ROS: reactive oxygen species
sgRNA: single‐guide RNA
TUNEL: terminal deoxynucleotidyl transferase biotin‐dUTP nick‐end labeling

Regucalcin (RGN) is a Ca^2+^‐binding protein that lacks the typical EF‐hand Ca^2+^‐binding domain 1, 2, and was also identified as senescence marker protein‐30, which is downregulated with aging in rat livers 3. Ca^2+^‐binding by human RGN was also confirmed in crystal structure and X‐ray diffraction analyses 4. Human and rat RGN genes are localized on the X chromosome and comprise seven exons and six introns 5, 6. Moreover, coding regions of vertebrate RGN are highly conserved 7 and the 5′‐flanking region of the RGN gene contains several consensus regulatory elements. Accordingly, the transcription factors AP‐1, NF1‐A1, RGPR‐p117, and β‐catenin have been shown to induce RGN gene promoter activity 8. RGN mRNA and protein expression was initially detected at high levels in liver and kidney tissues, and was then identified in various other tissues and was shown to be regulated by factors including calcium, calcitonin, parathyroid hormone, 1,25‐dihydroxyvitamin D_3_, insulin, tumor necrosis factor‐α, transforming growth factor‐β, estrogen, testosterone, aldosterone, dexamethasone, 17β‐estradiol, and 5α‐dihydrotestosterone 9, 10, 11.

Regucalcin is known to play multiple regulatory roles in mammalian cells. In particular, intracellular Ca^2+^ homeostasis is regulated by RGN via the activities of plasma membrane Ca^2+^‐ATPase, sarco‐/endoplasmic reticulum Ca^2+^‐ATPase, nuclear outer membrane Ca^2+^ pump, and mitochondrial Ca^2+^ uniporters in many cell types 12. RGN also regulates the intracellular Ca^2+^ signaling enzymes 5′‐nucleotidase, adenosine 5′‐triphosphatase, cAMP phosphodiesterase, protein kinase C, nitric oxide (NO) synthase, Ca^2+^/calmodulin protein kinase, phosphatase, and calpain, suggesting roles in various intracellular signaling pathways 13, 14, 15. Finally, RGN reportedly controls protein turnover by suppressing protein and nucleic acid synthesis and by activating proteases 13, 14, 15.

Several studies have shown that RGN suppresses cell proliferation through multiple signaling pathways 13, 14, 15. Furthermore, RGN may act as a suppressor protein that mitigates human carcinogenesis 16. Previously, we showed that RGN mRNA is downregulated in human tumor tissues *in vivo* 17, and subsequently, increased RGN gene expression is associated with prolonged survival of patients with pancreatic cancer, breast cancer, and hepatocarcinoma 18, 19, 20. Moreover, the overexpression of human RGN suppressed the proliferation of human pancreatic cancer PaCa‐2 cells, human breast cancer MDA‐MB‐231 cells, and human hepatocellular carcinoma HepG2 cells 18, 19, 20.

Alzheimer's disease is a neurodegenerative disease that is characterized by progressive declines in cognitive function, learning, and memory 21, 22. Excessive accumulation of amyloid‐β (Aβ) in brain cells is the key defining event in the pathogenesis of Alzheimer's disease, reflecting the neurotoxicity of Aβ, which is a major protein component of senile plaques 23, 24. Ca^2+^ signaling in neurons is central to neuronal functions, such as synaptic plasticity, learning, and memory. Hence, disruptions of Ca^2+^ transport through Ca^2+^ channels on plasma membranes, mitochondria, and the endoplasmic reticulum contribute to neurodegeneration and the development of Alzheimer's disease 25, 26. Aβ has been shown to elevate intracellular Ca^2+^ concentrations by inducing Ca^2+^ influx, leading to Ca^2+^‐mediated neurotoxicity 27, 28. The neurofibrillary pathogenesis of Alzheimer's disease involves tau hyperphosphorylation by Ca^2+^/calmodulin‐dependent protein kinase and cytoskeletal protein cleavage by Ca^2+^‐dependent protease calpains 29, 30, 31. Because RGN is a reported functional inhibitor of Ca^2+^/calmodulin‐dependent protein kinase 32 and calpains 33, 34, dysregulation of neuronal Ca^2+^ homeostasis during age‐related cognitive declines and neurodegenerative disease may be associated with decreased RGN expression 35.

Numerous studies indicate that Aβ‐mediated oxidative stress and mitochondrial damage are involved in the pathogenesis of Alzheimer's disease 36, 37, 38. Specifically, Aβ triggers oxidative stress in neuronal cells, in which mitochondrial dysfunction is accompanied by the production of reactive oxygen species (ROS) such as superoxide ($O_{2}^{-}$) and hydrogen peroxide (H_2_O_2_). 39, 40, 41. In addition, Aβ reportedly causes nitrosative stress in neuronal cells, in which inducible nitric oxide synthase (iNOS) produces reactive nitrogen species such as NO and peroxynitrite (ONOO^−^) 42, 43, 44. The consequent oxidative and/or nitrosative stress contributes to neuronal damage and leads to the formation of advanced glycation end products, advanced lipid peroxidation end products, and oxidized nucleic acids in Alzheimer's disease 38, 40, 45. Herein, we investigated the protective effects of RGN against Aβ‐induced neurotoxicity in neuronally differentiated PC12 cells and characterized the underlying neuroprotective mechanisms of action.

## Materials and methods

### Materials

The following materials from the indicated sources were used in this study: Dulbecco's modified Eagle's medium (DMEM), Dulbecco's PBS (DPBS), fetal bovine serum (FBS), horse serum (HS), penicillin, streptomycin, MitoSOX Red, tetramethylrhodamine methyl ester (TMRM) and 5‐(and‐6)‐chloromethyl‐2′,7′‐dichlorodihydrofluorescein diacetate acetyl ester (CM‐H_2_DCFDA) from Invitrogen (Carlsbad, CA, USA); rat nerve growth factor (NGF) and caspase florescence assay kit from R&D Systems, Inc. (Minneapolis, MN, USA); Aβ_25–35_ and Hoechst 33342 dye from Sigma‐Aldrich (St. Louis, MO, USA); pMXs‐puro retrovirus vector, Platinum‐E retrovirus packaging cells, and OxiSelect Nitrotyrosine ELISA kit from Cell Biolabs (San Diego, CA, USA); NeuroMag and ViroMag R/L viral gene delivery reagent from OZ Biosciences (Marseille, France); lipid peroxidation assay kit from BioVision (Milpitas, CA, USA); NO assay kit from Dojin (Kumamoto, Japan); Pierce BCA protein assay reagent kit from Thermo Scientific (Waltham, MA, USA); pGL4.32[*luc2P*/nuclear factor‐κB (NF‐κB)‐RE/Hygro] vector, pRL‐TK vector, and Dual‐Glo luciferase assay system from Promega (Madison, WI, USA); rabbit anti‐iNOS antibody from Cell Signaling Technology (Danvers, MA, USA); peroxidase‐conjugated donkey anti‐rabbit IgG antibody and enhanced chemiluminescent (ECL) western blotting detection reagents from Amersham Biosciences (Piscataway, NJ, USA); protease and phosphatase inhibitor cocktail tablets and *in situ* apoptosis detection kit from Roche (Mannheim, Germany); and lentiviral vectors containing a single‐guide RNA (sgRNA)/CRISPR/Cas9 All‐in‐One gene targeting system from Applied Biological Materials (Richmond, Canada).

### Cell culture and retrovirus infection

Pheochromocytoma PC12 cells were obtained from RIKEN BRC Cell Bank (Ibaraki, Japan) and then subcloned to generate more homogeneous populations. PC12 cells were cultured in DMEM supplemented with 5% heat‐inactivated FBS, 5% HS, 50 units·mL^−1^ penicillin, and 50 μg·mL^−1^ streptomycin in a humidified atmosphere of 5% CO_2_ and 95% air. Mouse RGN and β‐galactosidase (LacZ) cDNA were subcloned into the pMXs‐puro retrovirus vector. Vectors were transfected into Plat‐E retrovirus packaging cells, and PC12 cells were infected with retrovirus in the presence of ViroMag R/L viral gene delivery reagent. In RGN knockout manipulations using CRISPR/Cas9/sgRNA technology, sgRNA sequences targeting RGN were 5′‐GATTGCTGATCGAATCCCAT‐3′, 5′‐CGAGTGCAGCGAGTTGGTGT‐3′, and 5′‐AGGTACCATGGCTGAGGAAA‐3′, and a scramble sgRNA was used as a control sgRNA. A mixture of three types of lentiviruses expressing sgRNA against RGN was used to transduce PC12 cells. Retrovirus‐ or lentivirus‐infected cells were selected with puromycin (3 μm) for 3 weeks and were then used in experiments. PC12 cells were neuronally differentiated by treatment with NGF (100 ng·mL^−1^) in DMEM containing 1% HS. After selection by incubation with puromycin, neuronal differentiation of PC12 cells was induced by NGF (100 ng·mL^−1^) in DMEM containing 1% HS. Subsequently, Aβ_25–35_ peptide was dissolved in deionized water at 1 mm, applied to cells at 37 °C overnight to promote fibril formation, and then stored at −80 °C.

### Assay of apoptotic cells

DNA fragmentation was estimated using terminal deoxynucleotidyl transferase biotin‐dUTP nick‐end labeling (TUNEL) assays with an *in situ* apoptosis detection kit according to the manufacturer's instructions. Briefly, after treatment in chamber slides, cells were washed three times with ice‐cold DPBS and were fixed with 4% paraformaldehyde for 10 min. Cells were then washed twice in DPBS and were permeabilized using 0.1% Triton X‐100 in 0.1% sodium citrate for 10 min. Subsequently, cells were incubated with the TUNEL reaction mixture for 1 h at 37 °C in the dark. After washing twice with DPBS, nuclear counterstaining was performed with Hoechst 33342 dye (10 μg·mL^−1^) to determine total cell counts. Numbers of TUNEL‐positive cells per 500 cells were counted in randomly selected fields using a Zeiss fluorescence microscope (Carl Zeiss MicroImaging, Inc., Jena, Germany).

### Measurement of mitochondrial membrane potential

Mitochondrial membrane potential was measured using the fluorescent dye TMRM. Briefly, cells were treated in chamber slides and incubated in a medium containing TMRM (200 nm) for 20 min, washed with DPBS containing Hoechst 33342 dye (10 μg·mL^−1^) and then with DPBS alone, and then placed in phenol red‐free DMEM. Cells were then visualized using a Zeiss fluorescence microscope, and pictures were taken of random fields of view. Fluorescent intensities of TMRM were quantified using Zeiss software (Carl Zeiss MicroImaging, Inc., Jena, Germany).

### Assay of caspase activity

Caspase activities were measured using fluorescent substrates as described previously 46 with minor modifications. Briefly, caspase‐9 and caspase‐3 activities were measured using a caspase fluorescence assay kit according to the manufacturer's instructions. Briefly, after treatment on 24‐well dishes, cells were washed with ice‐cold DPBS and were lysed in caspase lysis buffer for 15 min on ice. Cells were then centrifuged at 15 000 ***g*** for 15 min at 4 °C, and protein concentrations of supernatants were determined using micro‐BCA protein assay reagent kits. Supernatants containing 50 μg of protein were then incubated with 50 μm LEHD‐AFC (caspase‐9 substrate) or DEVD‐AFC (caspase‐3 substrate) for 1 h, and caspase activities were assayed using fluorometric determinations of the hydrolyzed products with a Perkin Elmer microplate spectrofluorometer (EnSpire, Norwalk, CT, USA) at excitation and emission wavelengths of 400 and 505 nm, respectively. Enzyme activities were expressed as fluorescence intensities in arbitrary units (a.u.) per mg of total protein.

### Measurement of mitochondrial and intracellular ROS

Mitochondrial ROS generation was visualized using the fluorescent dye MitoSOX Red, which is sensitive to $O_{2}^{-}$. Intracellular ROS generation was also visualized using the fluorescent dye CM‐H_2_DCFDA, which is particularly sensitive to H_2_O_2_ among various ROS. In these experiments, cells were treated in chamber slides, then incubated in medium containing MitoSOX Red (5 μm) or CM‐H_2_DCFDA (5 μm) for 30 min, washed with DPBS containing Hoechst 33342 dye (10 μg·mL^−1^) and then with DPBS alone, and then placed in phenol red‐free DMEM. Cells were visualized using a Zeiss fluorescence microscope, and pictures were taken of randomly selected fields of view. Fluorescent intensities of MitoSOX and CM‐H_2_DCFDA were quantified using Zeiss software.

### Assay of lipid peroxidation

Oxidative damage was assayed by measuring malondialdehyde concentrations using a lipid peroxidation assay kit according to the manufacturer's protocol. Briefly, after treatment, cells were washed with DPBS, harvested by trypsinization, and then sonicated for 20 s in malondialdehyde lysis buffer. After centrifugation at 13 000 ***g*** for 10 min, supernatants were incubated with thiobarbituric acid at 95 °C for 1 h and were then cooled to room temperature. Subsequently, samples were transferred to 96‐well plates for fluorometric analyses with a Perkin Elmer microplate spectrofluorometer. Protein concentrations of supernatants were determined using a micro‐BCA protein assay reagent kit. Malondialdehyde contents were normalized to mg of total protein.

### Measurement of NO production

Nitric oxide production was estimated from nitrite contents that were detected using NO assay kits with the Griess reaction according to the manufacturer's instructions. After treatment on 24‐well dishes, culture media were mixed with Griess reagent and incubated for 20 min at room temperature. Subsequently, absorbances of mixtures were determined at 540 nm using a Perkin Elmer microplate spectrofluorometer.

### Immunoblot analysis of iNOS expression

After treatment on 35‐mm dishes, cells were lysed in RIPA lysis buffer containing the protease inhibitor mixture. Lysed cells were then centrifuged at 15 000 ***g*** for 15 min at 4 °C, and protein concentrations of supernatants were determined using micro‐BCA protein assay reagent kits. Supernatants containing 50 μg of protein were then boiled in Laemmli sample buffer containing 5% 2‐mercaptoethanol. Proteins were resolved using 12% sodium dodecyl sulfate/polyacrylamide gel electrophoresis and were transferred to polyvinylidene difluoride membranes. After blocking for 1 h in buffer containing 20 mm Tris/HCl (pH 7.6), 100 mm NaCl, 0.1% Tween‐20 (TBS‐T), and 3% skim milk, membranes were incubated with rabbit anti‐iNOS antibody (1 : 100) in TBS‐T containing 1% skim milk and then with peroxidase‐conjugated donkey anti‐rabbit IgG antibody in the same buffer. Bound antibody was visualized using the ECL system. Blots were then stripped and reprobed with anti‐β‐actin antibody to use β‐actin as a loading control.

### Assay of transcriptional activity of nuclear factor‐κB (NF‐κB)

Nuclear factor‐κB transcriptional activity was determined by a reporter gene assay as described previously 46 with minor modifications. Briefly, the transcriptional activity of NF‐κB was measured after transfecting cells with the pRL‐TK vector (0.125 μg per well) and the NF‐κB‐TA‐Luc vector (0.5 μg per well) or the molar equivalent of the pTA‐Luc vector (negative control). Transfection was performed with the NeuroMag reagent according to the manufacturer's instructions. The pRL‐TK vector contains the *Renilla* luciferase gene under the control of the minimum promoter from herpes simplex virus thymidine kinase, and was used as an internal control for differences in transfection efficiencies and cell numbers. After transfection, cells were treated in DMEM with or without Aβ for various times. Transfected cells were then lysed, and luciferase activities of lysates were measured using dual‐luciferase assays according to the manufacturer's instructions. Reporter gene activity was expressed as firefly luciferase activity of the NF‐κB‐TA‐Luc vector divided by *Renilla* luciferase activity of the pRL‐TK vector. Luciferase activity of the pTA‐Luc vector was subtracted from that of the NF‐κB‐TA‐Luc vector.

### Measurements of nitrotyrosine contents

Intracellular ONOO^−^ formation was investigated as an indicator of nitrosative stress by determining intracellular 3‐nitrotyrosine contents using OxiSelect Nitrotyrosine ELISA kits according to the manufacturer's instructions. Briefly, cells were treated on 24‐well dishes and were then washed with ice‐cold DPBS and lysed in RIPA buffer containing protease and phosphatase inhibitor mixtures for 15 min on ice. After centrifugation at 15 000 ***g*** for 15 min at 4 °C, supernatants were collected and nitrotyrosine contents were normalized to mg of total protein, which were determined using micro‐BCA protein assay reagent kits.

### Statistical analysis

The significance of differences was estimated with Student's *t*‐test using GraphPad Prism software (GraphPad software, Inc., San Diego, CA, USA). A *P* value of < 0.05 was considered significant.

## Results and Discussion

**Figure 1:**
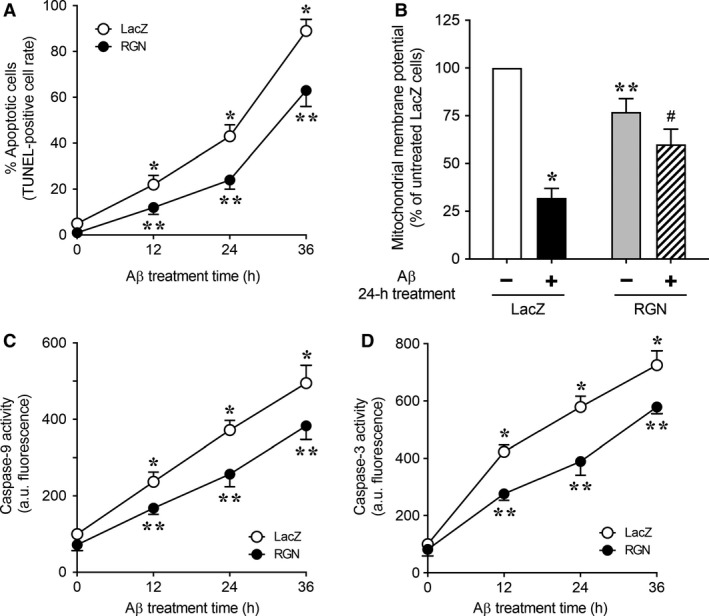
Regucalcin attenuates Aβ‐induced apoptosis by activating the mitochondrial caspase pathway in neuronally differentiated PC12 cells. NGF‐differentiated PC12 cells overexpressing β‐galactosidase (LacZ) or RGN were treated with 25 μm Aβ for the indicated periods. (A) Cells were incubated with the TUNEL reaction mixture to determine rates of apoptosis and then stained with the Hoechst 33342 nuclear stain. TUNEL‐positive apoptotic nuclei were identified using a fluorescent microscope. (B) Mitochondrial membrane potential was measured using the fluorescent probe TMRE. (C,D) Caspase‐9‐mediated cleavage of LEHD‐AFC and caspase‐3‐mediated cleavage of DEVD‐AFC were estimated in cell lysates using fluorometric assays with specific substrates. Data are expressed as mean a.u. of fluorescence/mg protein ± standard errors of the mean (SE) from three independent experiments performed in triplicate. (A) *P < 0.05, Aβ‐treated LacZ cells versus untreated LacZ cells at time 0; **P < 0.05, Aβ‐treated RGN cells versus Aβ‐treated LacZ cells at the same time point; (B) *P < 0.05, Aβ‐treated LacZ cells versus untreated LacZ cells; **P < 0.05, untreated RGN cells versus untreated LacZ cells; #*P* < 0.05, Aβ‐treated RGN cells versus Aβ‐treated LacZ cells; (C and D) *P < 0.05, Aβ‐treated LacZ cells versus untreated LacZ cells at time 0; **P < 0.05, Aβ‐treated RGN cells versus Aβ‐treated LacZ cells at the same time point.

After retrovirus‐mediated gene transfer of RGN or LacZ, infected cells were selected using puromycin treatments. Subsequently, we determined whether RGN modulates Aβ‐induced apoptosis in neuronally differentiated PC12 cells by measuring apoptotic responses to Aβ using TUNEL staining analyses (Fig. 1A). In these experiments, numbers of apoptotic cells in LacZ‐overexpressing control cells were significantly increased after Aβ treatment in a time‐dependent manner. Compared with LacZ control cells, numbers of Aβ‐induced apoptotic cells were fewer among RGN‐overexpressing cells, suggesting that RGN protects against Aβ‐induced apoptosis in neuronally differentiated PC12 cells.

Previous studies show that Aβ reduces mitochondrial membrane potential in neuronal cells, indicating mitochondrial dysfunction under these conditions 36, 37, 38. Accordingly, we examined the effects of RGN overexpression on Aβ‐induced loss of mitochondrial membrane potential in neuronally differentiated PC12 cells (Fig. 1B). We confirmed Aβ‐induced decreases in mitochondrial membrane potential in LacZ‐overexpressing control cells and observed slightly lower mitochondrial membrane potential in untreated RGN‐overexpressing cells than in untreated LacZ‐overexpressing cells. However, following treatment with Aβ, RGN‐overexpressing cells had significantly higher mitochondrial membrane potential than LacZ‐overexpressing cells, and no significant differences in mitochondrial DNA copy numbers were observed (data not shown). These results indicate that RGN blocks the induction of mitochondrial dysfunction by Aβ, as characterized by loss of mitochondrial membrane potential. It is widely accepted that Aβ‐mediated loss of mitochondrial membrane potential promotes mitochondrial apoptosis, during which cytoplasmic release of cytochrome *c* activates caspase‐9 and downstream caspase‐3 47. Thus, we examined time‐dependent changes in caspase activity in neuronally differentiated PC12 cells overexpressing LacZ or RGN following treatment with Aβ (Fig. 1C,D). In these experiments, LacZ‐overexpressing cells showed increased caspase‐9 and caspase‐3 activities following treatment with Aβ, and overexpression of RGN resulted in significant decreases in Aβ‐induced activities of both caspases when compared with LacZ‐overexpressing control cells. Taken together, these data indicate that RGN attenuates Aβ‐induced mitochondrial apoptosis by maintaining mitochondrial membrane potential.

**Figure 2:**
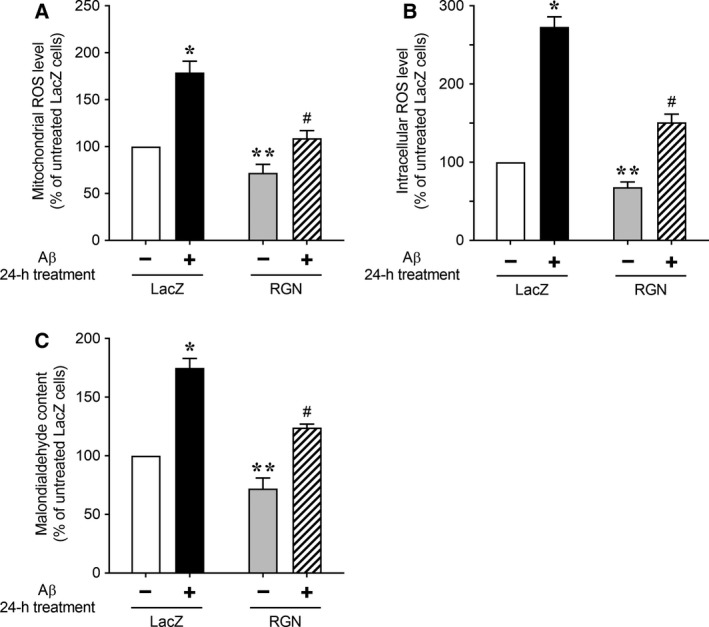
RGN attenuates Aβ‐induced oxidative damage in neuronally differentiated PC12 cells. NGF‐differentiated PC12 cells overexpressing LacZ or RGN were treated with 25 μm Aβ for 24 h. (A) Mitochondrial ROS generation was detected using the fluorescent ($O_{2}^{-}$) probe MitoSOX Red. (B) Intracellular ROS generation was detected using fluorescent hydrogen peroxide indicator CM‐H_2_ DCFDA. (C) Oxidative damage was estimated by measuring malondialdehyde contents as an index of lipid peroxidation. Data are presented as means ± SE of three independent experiments performed in triplicate. *P < 0.05, Aβ‐treated LacZ cells versus untreated LacZ cells; **P < 0.05, untreated RGN cells versus untreated LacZ cells; ^#^ *P* < 0.05, Aβ‐treated RGN cells versus Aβ‐treated LacZ cells.

Amyloid‐β‐mediated disruptions of mitochondrial function are prominent early events in Alzheimer's disease. Moreover, Aβ exposure of neuronal cells has been shown to increase intracellular ROS production, which has been strongly associated with mitochondrial dysfunction 39, 40, 41. Mitochondria are the main source of intracellular ROS, likely reflecting production of $O_{2}^{-}$ 48 and subsequent conversion into H_2_O_2_ spontaneously or by superoxide dismutase 48. Hence, we examined mitochondrial ROS production in LacZ‐ or RGN‐overexpressing cells using the fluorescent dye MitoSOX Red, which is rapidly oxidized by $O_{2}^{-}$ (Fig. 2A). In these studies, control cells overexpressing LacZ showed significant increases in mitochondrial ROS after treatment with Aβ. Moreover, untreated RGN‐overexpressing cells had significantly lower basal levels of mitochondrial ROS than those overexpressing LacZ. Following treatment with Aβ, RGN‐overexpressing cells exhibited significant decreases in mitochondrial ROS production compared with LacZ‐overexpressing control cells, suggesting that RGN attenuates basal and Aβ‐induced mitochondrial ROS production. In further assays using the intracellular ROS indicator CM‐H_2_DCFDA, we measured differences in intracellular ROS levels in LacZ‐ or RGN‐overexpressing cells exposed to Aβ (Fig. 2B) and confirmed that RGN overexpression decreases basal and Aβ‐induced intracellular ROS production. These results suggest that RGN attenuates basal and Aβ‐induced intracellular ROS contents by regulating mitochondrial ROS production.

Regucalcin has been shown to be localized to mitochondria 49, and previous electron microscopy analyses showed abnormally enlarged mitochondria with indistinct cristae in hepatocytes from RGN knockout mice, compared with those in wild‐type mice 50. These reports imply that RGN plays an important role in the maintenance of mitochondrial function. Normal mitochondria mediate redox signaling by releasing ROS from the electron transport chain, and overexpression of RGN decreased mitochondrial ROS production under basal conditions [see Fig. 2A; LacZ (−) versus RGN (−)]. Hence, RGN may preserve electron transport chain activity, leading to a lower mitochondrial membrane potential under basal conditions [Fig. 1B; LacZ (−) versus RGN (−)]. These data suggest that RGN protects neuronal cells from Aβ‐mediated neurotoxicity by ameliorating mitochondrial ROS generation.

Amyloid‐β‐induced excessive ROS generation reportedly causes oxidative damage to cell components including lipids, proteins, and DNA and thereby disrupts neuronal cell function 38, 40, 45. Thus, to determine whether RGN attenuates Aβ‐induced oxidative damage, we determined ROS‐mediated lipid peroxidation according to concentrations of the end product malondialdehyde (Fig. 2C). Significantly increased levels of lipid peroxidation were observed in Aβ‐treated cells overexpressing LacZ relative to untreated LacZ‐overexpressing control cells. However, upon treatment with Aβ, RGN‐overexpressing cells had significantly lower malondialdehyde levels than LacZ‐overexpressing cells, indicating that RGN protects against Aβ‐mediated lipid peroxidation.

Published *in vitro* studies show that RGN is cytoprotective against apoptotic cell death induced by tumor necrosis factor‐α, lipopolysaccharide, transforming growth factor‐β1, and thapsigargin 10, 11. In agreement, previous *ex vivo* studies of seminiferous tubules from transgenic rats showed protective effects of RGN overexpression against tert‐butyl hydroperoxide‐ and cadmium‐induced oxidative stress in rat testis 51. Moreover, *in vivo* studies with RGN knockout mice showed high susceptibility to age‐ and smoking‐related oxidative stress in lungs 52. These RGN knockout mice also had elevated oxidative stress in the brain 53. Thus, RGN likely plays important roles in cell defenses against oxidative stress. Furthermore, RGN expression is decreased with aging in the cerebral cortex and hippocampus 54, further indicating that age‐related decreases in RGN expression contribute to Aβ‐mediated oxidative stress and neurotoxicity in Alzheimer's disease.

**Figure 3:**
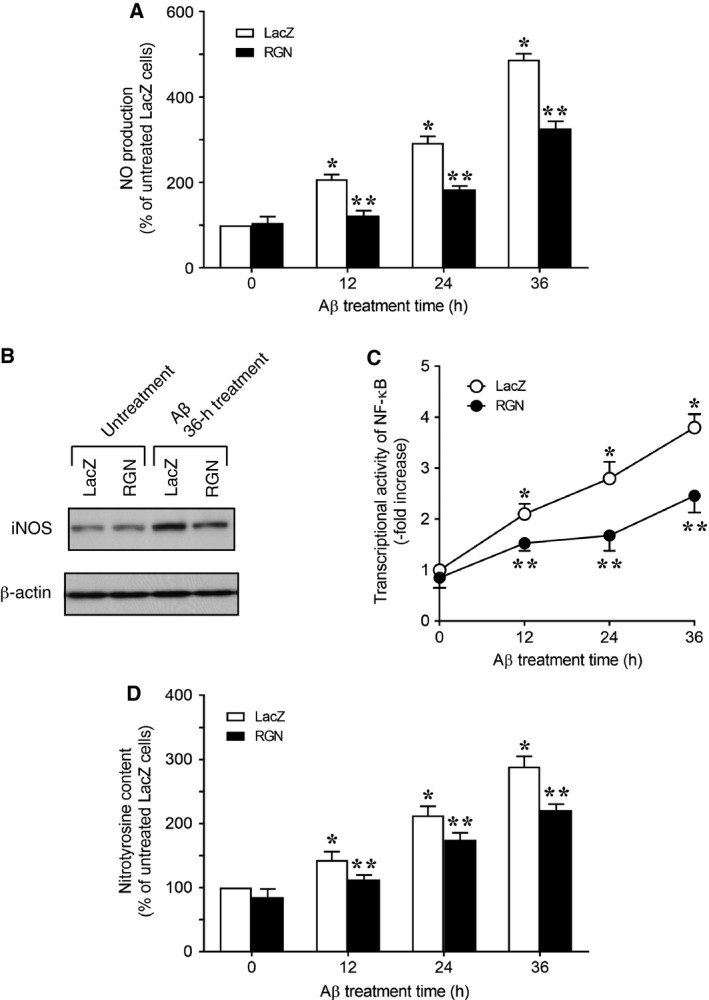
RGN attenuates Aβ‐induced nitrosative damage in neuronally differentiated PC12 cells. NGF‐differentiated PC12 cells overexpressing LacZ or RGN were treated with 25 μm Aβ for the indicated time periods. (A) NO contents of medium were measured using the Griess method. (B) iNOS protein expression was determined using western blotting with a specific antibody. Actin expression was used as a protein loading control. (C) Cells were transiently cotransfected with a NF‐κB‐responsive reporter vector and the internal control vector pRL‐TK. After 12 h, transfected cells were treated with Aβ for the indicated periods and were then lysed for NF‐κB reporter assays with a dual‐luciferase reporter assay system. Data are presented as fold increases compared with luciferase activity at 0 h in LacZ cells, which was set at 1.0. (D) Nitrosative damage was estimated by measuring content of nitrotyrosine, which indicates the nitration of tyrosine by ONOO ^−^ generated from NO and $O_{2}^{-}$. Data are presented as means ± SE of three independent experiments performed in triplicate. (A, C, and D) *P < 0.05, Aβ‐treated LacZ cells versus untreated LacZ cells at time 0; **P < 0.05, Aβ‐treated RGN cells versus Aβ‐treated LacZ cells at the same time point.

In addition to oxidative damage, nitrosative damage due to overproduction of NO has been associated with the pathophysiology of Alzheimer's disease 55, 56. In particular, the exposure of neuronal cells to Aβ led to NO overproduction by upregulating iNOS 57, 58, 59, 60; in other studies, Aβ caused NO‐mediated nitrosative damage in neuronal cells 42, 43, 44. Accordingly, we examined the effects of RGN overexpression on NO production in neuronally differentiated PC12 cells in the presence of Aβ (Fig. 3A) and showed significantly greater NO production following treatment of LacZ‐overexpressing control cells with Aβ. In separate experiments, RGN‐overexpressing cells exhibited significant decreases in NO production following exposure to Aβ when compared with LacZ‐overexpressing control cells.

Amyloid‐β‐induced NO production was previously shown to follow upregulation of iNOS mRNA by the transcription factor NF‐κB 60. Hence, to further investigate the roles of RGN in iNOS expression and NF‐κB activity, we examined whether iNOS protein levels and NF‐κB activity are altered by overexpression of RGN (Fig. 3B,C). In these experiments, exposure of LacZ‐expressing control cells to Aβ resulted in increases in both iNOS expression and NF‐κB activity. In contrast, RGN overexpression led to significantly lower Aβ‐induced iNOS expression and NF‐κB activity than in LacZ‐overexpressing control cells, indicating that RGN inhibits Aβ‐induced NO overproduction by attenuating NF‐κB‐mediated iNOS expression.

Intracellular NO reacts rapidly with $O_{2}^{-}$ to produce the powerful oxidant ONOO^−^, which is a major causal factor in NO‐mediated neurotoxicity 42, 43, 44. Because ONOO^−^ selectively nitrates tyrosine residues of proteins 45, 56, we estimated the effects of RGN overexpression on Aβ‐induced intracellular nitrotyrosine levels. These experiments showed increased intracellular nitrotyrosine levels in Aβ‐treated LacZ‐overexpressing control cells and significantly attenuated Aβ‐induced nitrotyrosine production in RGN‐overexpressing cells. Taken together, these results suggest that RGN reduces Aβ‐induced nitrosative cell damage by reducing the production of reactive nitrogen species such as NO and ONOO^−^.

**Figure 4:**
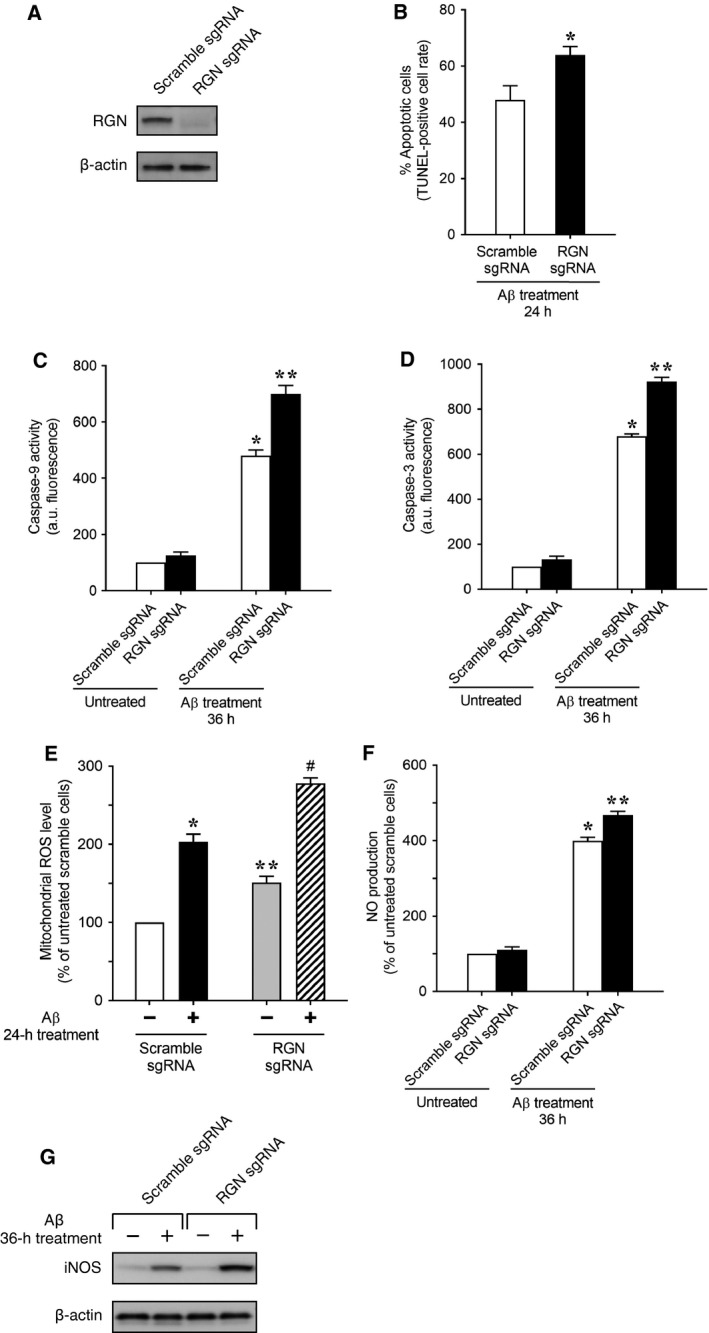
RGN‐deficient PC12 neuron‐like cells become vulnerable to Aβ toxicity. A lentivirus‐mediated CRISPR/Cas9 system was used to generate PC12 cells, in which the RGN gene is knocked out. PC12 cells were infected with lentiviruses harboring RGN sgRNA or control scramble sgRNA, selected with puromycin, and then neuronally differentiated using treatments with NGF. (A) Knockdown efficiency was examined by western blotting using β‐actin as a loading control. (B–F) Upon treatment with Aβ, apoptosis assay, caspase assay, mitochondrial ROS measurement, NO assay, and iNOS western blotting were performed as described in the legends to Figs 1A,C,D, 2A and 3A,B, respectively. Data are presented as means ± SE of three independent experiments performed in triplicate. (B) *P < 0.05, Aβ‐treated RGN sgRNA cells versus Aβ‐treated scramble sgRNA cells; (C,D) *P < 0.05, Aβ‐treated scramble sgRNA cells versus untreated scramble sgRNA cells; **P < 0.05, Aβ‐treated RGN sgRNA cells versus Aβ‐treated scramble sgRNA cells; (E) *P < 0.05, Aβ‐treated scramble sgRNA cells versus untreated scramble sgRNA cells; **P < 0.05, untreated RGN sgRNA cells versus untreated scramble sgRNA cells; ^#^ *P* < 0.05, Aβ‐treated RGN sgRNA cells versus Aβ‐treated scramble sgRNA cells; (F) *P < 0.05, Aβ‐treated scramble sgRNA cells versus untreated scramble sgRNA cells; **P < 0.05, Aβ‐treated RGN sgRNA cells versus Aβ‐treated scramble sgRNA cells.

To further investigate the protective effect of RGN on Aβ toxicity in PC12 neuron‐like cells, we used the CRISPR/Cas9 technique to disrupt the RGN gene in PC12 cells and then examined the sensitivity of RGN knockout PC12 cells to Aβ. RGN expression was induced by the treatment of PC12 cells with NGF (data not shown). As shown in Fig. 4A, the immunoblot analysis of neuronally differentiated PC12 cells expressing scramble sgRNA confirmed the presence of endogenous RGN protein. In contrast, endogenous RGN protein was not detected in neuronally differentiated PC12 cells expressing sgRNA targeting RGN, indicating successful RGN gene knockout in PC12 cells. As shown in Fig. 4B, the cells expressing sgRNA targeting RGN potentiated Aβ‐induced increases in numbers of apoptotic cells when compared with control cells expressing scramble sgRNA. In addition, because we found that Aβ activates the caspase‐9/3 cascade via mitochondrial dysfunction, as indicated by decreases in mitochondrial membrane potential (see Fig. 1B–D), we examined the effects of RGN loss on the Aβ‐induced activation of the caspase‐9/3 cascade (Fig. 4C,D). The cells expressing sgRNA targeting RGN exhibited significant increases in the Aβ‐induced activity of caspase‐9 and caspase‐3 when compared with control cells expressing scramble sgRNA, suggesting that RGN deficiency exacerbates Aβ‐induced apoptosis by activating the caspase‐9/3 cascade via mitochondrial dysfunction. Moreover, because we found that Aβ increased mitochondrial ROS production, which leads to an increase in intracellular ROS level and subsequent oxidative damage (see Fig. 2A–C), we examined the effect of RGN loss on Aβ‐induced increases in mitochondrial ROS production (Fig. 4E). Under untreated conditions, the cells expressing sgRNA targeting RGN showed significant increases in mitochondrial ROS production compared with scramble sgRNA‐expressing control cells (see Fig. 4E; Aβ (−)/scramble sgRNA versus Aβ (−)/RGN sgRNA), suggesting that RGN controls mitochondrial ROS generation under basal conditions. Upon treatment with Aβ, mitochondrial ROS production in RGN sgRNA‐expressing cells was significantly increased relative to that in scramble sgRNA‐expressing control cells (see Fig. 4E; Aβ (+)/scramble sgRNA versus Aβ (+)/RGN sgRNA), suggesting that RGN deficiency enhances Aβ‐induced oxidative stress by increasing mitochondrial ROS production.

Furthermore, because we found that Aβ caused nitrosative cell damage by promoting NO production via NF‐κB activation‐mediated iNOS expression (see Fig. 3A–D), we examined the effect of RGN loss on Aβ‐induced NO production and iNOS expression (Fig. 4F,G). The exposure of cells expressing sgRNA targeting RGN to Aβ caused significant increases in NO production and iNOS expression compared with that in control cells expressing scramble sgRNA. These results suggest that RGN deficiency potentiates Aβ‐mediated nitrosative stress by increasing iNOS‐mediated NO production. Taken together, the knockout data described above suggest that RGN protects PC12 neuron‐like cells from Aβ‐induced apoptosis based on oxidative and nitrosative stresses. Thus, consistent with our findings from RGN overexpression experiments, the effects of RGN knockout provide further evidence of the protective function of RGN against Aβ toxicity.

In the present study, we demonstrate that RGN attenuates the susceptibility of neuronally differentiated PC12 cells to Aβ‐induced apoptosis. We also showed that RGN prevents oxidative and nitrosative Aβ toxicity in neuronally differentiated PC12 cells. Thus, RGN may play an important protective role in neurons of patients with Alzheimer's disease, and our findings provide novel insights into cellular defense mechanisms against Aβ neurotoxicity. Further *in vivo* studies are required to determine whether RGN protects neuronal cells from the Aβ toxicity that occurs in Alzheimer's disease. Thus, because we previously generated transgenic rats overexpressing RGN 61, it will be valuable to determine whether RGN transgenic rats are more or less sensitive than control rats to intracerebroventricular injections of Aβ. In addition, the present data from RGN overexpression experiments warrant determinations of whether virus‐mediated RGN gene transfer reverses pathologic changes and behavioral deficits in mouse models of Aβ‐mediated Alzheimer's disease. Furthermore, the present RGN knockout data indicate the value of experiments designed to determine whether brain‐specific RGN‐deficient mice exhibit neuronal vulnerability to Aβ toxicity. Finally, a previous report showed that the natural product compound EUK4010 inhibits Aβ‐induced neuronal cell damage and attenuates Aβ‐mediated suppression of RGN 62. Taken together, these data warrant consideration of RGN as a therapeutic target for Aβ‐mediated neuronal toxicity. Specifically, further investigations of the protective effects of RGN against Aβ neurotoxicity may lead to the development of novel neuroprotective strategies for the treatment of Alzheimer's disease.

## Author contributions

TM, MK, YS, MK, and KH performed the experiments, SK and CT constructed retrovirus expression system, and TM and MY designed the experiment and discussed with NK. MY provided comments pertaining to the manuscript. TM wrote the manuscript. All authors read and commented on the manuscript.
